# Embolization of a Jejunal Artery Pseudoaneurysm via Collateral Vessels

**DOI:** 10.1155/2015/465143

**Published:** 2015-12-20

**Authors:** Romain Breguet, Lawrence F. Pupulim, Sylvain Terraz

**Affiliations:** Department of Radiology, University Hospitals of Geneva, Rue Gabrielle-Perret-Gentil 4, 1205 Geneva, Switzerland

## Abstract

Visceral artery pseudoaneurysms are rare and only few cases have been reported. They are considered to be life threatening in case of rupture. Rapid treatment is mandatory and endovascular procedure is recommended as the treatment of choice. Occasionally, endovascular approach is difficult to achieve, owing to unusual vascular anatomy. Whenever it is the case, an alternative method has to be considered. We report the case of a jejunal artery pseudoaneurysm that required an access via collateral vessels to accomplish complete occlusion in a 34-year-old woman who presented with a sudden epigastric pain 14 days after a cephalic duodenopancreatectomy.

## 1. Introduction 

Jejunal artery pseudoaneurysms are known to be rare and only few cases have been reported [1, 2]. As other visceral pseudoaneurysms, they are considered life threatening vascular lesions in case of rupture. Rapid treatment of these lesions is mandatory and endovascular procedure has been recently recommended as the treatment of choice. Occasionally, endovascular approach is difficult to achieve, owing to unusual vascular anatomy or pseudoaneurysm location. Whenever it is the case, an alternative method has to be considered and the aim of this report is to stress the idea that collateral vessels of the mesenteric arterial arcade should be explored, when proximal direct superselective catheterization of jejunal branch pseudoaneurysms is difficult.

## 2. Case Report

**Figure 1:**
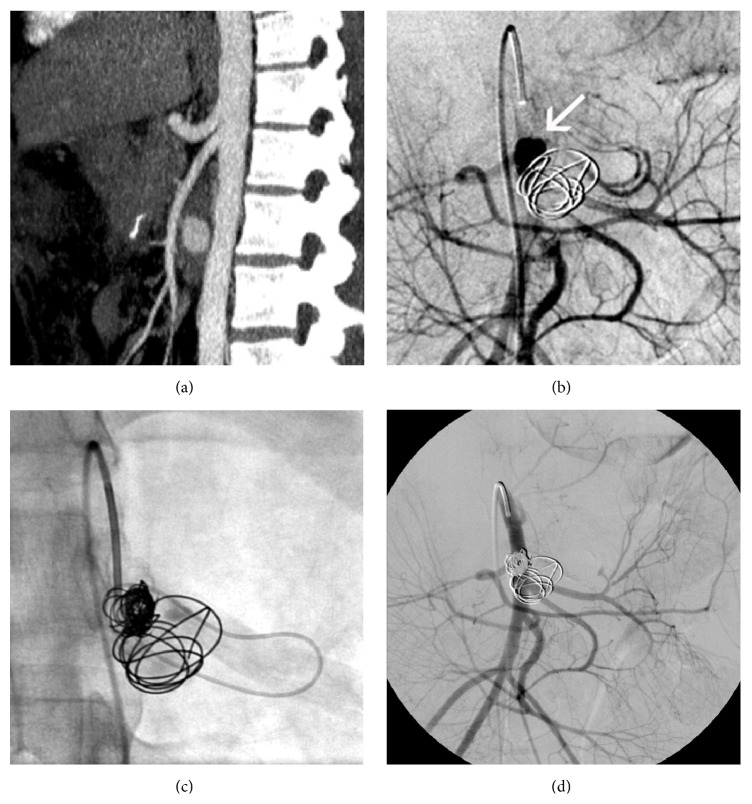
34-year-old woman presenting a jejunal artery pseudoaneurysm treated by transcatheter embolization via collaterals vessels. (a) Sagittal contrast enhanced CT image showing a pseudoaneurysm emerging from the first SMA branch (jejunal artery) just after its origin. (b) Digital angiogram obtained after coil embolization shows that pseudoaneurysm is still partially patent (white arrow) requiring further embolization; however subsequent direct catheterization was not possible. The image shows a collateral vascular arc between the first and the second jejunal arteries. (c) The collateral vascular arc was used to obtain pseudoaneurysm catheterization. (d) Complete embolization was finally obtained.

A 34-year-old female patient presented a jejunal artery pseudoaneurysm after cephalic duodenopancreatectomy (CDP), which required an access via collateral vessels to accomplish complete occlusion. The patient was addressed to our institution for a long-lasting history of idiopathic chronic pancreatitis and was submitted to CPD with the intention to treat a severe recurrent abdominal pain syndrome associated to stenosis of the main pancreatic duct. During the recovery period, pulmonary embolism was diagnosed and the patient received full dose anticoagulant therapy. On day 14, she presented acute epigastric pain and abdominal CT imaging demonstrated a well-defined 1.7 cm pseudoaneurysm of the first jejunal branch close to the origin of the superior mesenteric artery (SMA) (Figure 1(a)). After multidisciplinary meeting, transcatheter coil embolization was considered as the optimal treatment option. This procedure was performed in the interventional radiology suite through a 5-French sheath percutaneously placed in the right common femoral artery. After SMA catheterization, direct access into the pseudoaneurysm sac was obtained using a 2.7-F microcatheter (Progreat; Terumo, Tokyo, Japan) and one electrolytically detachable coil (GDC 14 mm–30 cm, Boston Scientific Corporation, Natick, MA, USA) was used to occlude the pseudoaneurysm. During coil release, the direct access into the pseudoaneurysm was lost, even after many attempts. Subsequent images showed that only incomplete filling was obtained and further embolization was necessary (Figure 1(b)). Analysis of the previous angiographic images showed that the efferent artery branch of the aneurysm was in communication with a collateral vascular arch between first and second jejunal arteries, which could permit an access through the opposite side. A microcatheter was successfully and rapidly placed into the pseudoaneurysm using this route (Figure 1(c)). Two more electrolytically detachable coils were released, allowing a complete occlusion of the pseudoaneurysm (Figure 1(d)). The clinical outcome was uneventful and no residual symptoms were observed after the procedure. The follow-up CT scan showed a good exclusion of the pseudoaneurysm and no other complications.

## 3. Discussion 

The most frequent sites of visceral pseudoaneurysms are splenic, hepatic, SMA, and celiac trunk. Other sites, such as gastroduodenal, pancreatic-duodenal, inferior mesenteric, and jejunal artery, are rare. Surgical resection, ligation, and bypass were historically the treatments of choice for visceral pseudoaneurysms, but nowadays current therapeutic modalities consist of percutaneous transcatheter techniques such as embolization [3] and prosthetic stent-graft. As reported in the literature, surgical treatment should be chosen only for cases diagnosed during laparotomy or in cases of prior unsuccessful endovascular treatment [3].

The technique of transcatheter superselective coil embolization may be sometimes complex to obtain because of vascular anatomy, tortuous nature of vessels, or problematic superselective catheterization. Sometimes, a second direct catheterization of the pseudoaneurysm is not possible to perform, even after several attempts, as experienced by the authors with this case. At this situation, some possibilities should be considered, such as placement of stent-graft, entire vessel embolization, percutaneous direct pseudoaneurysm puncture, or surgical approach. Placement of stent-graft may be problematic in pseudoaneurysms that originate from SMA or jejunal branches. Since it could result in occlusion of other normal vascular branches next to the pseudoaneurysm by extension of the stent-graft, the risk of intestinal ischemia is significantly increased. Furthermore, stent-graft can be complicated by occlusion and it usually requires prolonged postprocedure anticoagulation therapy. Alternatively, direct percutaneous puncture of the pseudoaneurysm followed by injection of cyanoacrylate or thrombin can be obtained under ultrasound or CT guidance. However, this particular technique may be difficult due to the localization of such pseudoaneurysms and interposition of intestinal loops. In this case, the pseudoaneurysm was located posterior to the main trunk of the SMA and, thus, difficult to reach by direct percutaneous approach.

The well-known procedure usually performed to treat splenic pseudoaneurysms involves isolation and exclusion of the afferent and efferent arteries close to the pseudoaneurysm. This method is not suitable for a pseudoaneurysm of the SMA, because of the high risk of intestinal ischemia.

In this case, superselective catheterization of the pseudoaneurysm by proximal approach was initially obtained, but complete embolization was not achieved (Figure 1(b)). Subsequently, superselective catheterization was achieved only by using a mesenteric arterial arcade (Figure 1(c)). The authors do not know exactly the reason why a second direct superselective catheterization was not achieved. They hypothesize that hemodynamic alterations of the pseudoaneurysm occurred after the first coil deployment and modifications of the pseudoaneurysm collar permitted an easier access by a retrograde approach.

Embolization of a splenic aneurysm via collateral vessels was already reported [4]. These authors used cyanoacrylate to the embolization of the efferent portion of splenic artery. To our best knowledge, the catheterization of a jejunal artery branch pseudoaneurysm via collateral vessels has not been already reported. The authors believe that catheterization of a pseudoaneurysm via collateral vessels may be challenging sometimes. Also, vascular anatomy may give the impression that access by collaterals is more difficult to obtain than the direct access. Nevertheless, in this case it was feasible and considerably easier to obtain.

## 4. Conclusion

The present case report is exquisite due to the rarity of jejunal artery pseudoaneurysms and the unusual approach used to occlude this vascular anomaly. However, it shows that when the initial treatment strategy has to be reconsidered during the procedure, in order to avoid an emergency laparotomy with related high morbidity rate, transcatheter embolization via collaterals vessels should be explored.

## References

[1] Chenoweth J. L., Patel B. K., Khosla A. (1998). Delayed traumatic pseudoaneurysm of a jejunal artery: a case report. *Vascular Surgery*.

[2] Bavunoglu I., Ayan F., Karabicak I. (2006). Selective jejunal artery pseudoaneurysm embolization in a patient with massive gastrointestinal bleeding due to intestinal tuberculosis. *Journal of Emergency Medicine*.

[3] Laganà D., Carrafiello G., Mangini M. (2006). Multimodal approach to endovascular treatment of visceral artery aneurysms and pseudoaneurysms. *European Journal of Radiology*.

[4] Ishimaru H., Murakami T., Matsuoka Y. (2004). Butyl 2-cyanoacrylate injection to occlude splenic artery distal to large splenic aneurysm after proximal coil embolization. *American Journal of Roentgenology*.

